# Perioperative Severe Hypotension in a Patient with Multiple Endocrine Neoplasia Type IIb and Bilateral Adrenalectomies: Time to Review the Evidence for Stress Dose Steroids

**DOI:** 10.1155/2016/8153296

**Published:** 2016-11-09

**Authors:** Jens Tan, Acsa Zavala, Katherine B. Hagan, Antoinette Van Meter, Uduak Ursula Williams, Wei Zhang, Pascal Owusu-Agyemang

**Affiliations:** Department of Anesthesiology and Perioperative Medicine, University of Texas MD Anderson Cancer Center, Houston, TX, USA

## Abstract

Multiple endocrine neoplasia type IIb (MEN IIb) is an endocrine disorder which can manifest with tumors such as pheochromocytomas and neuromas. We present the case of a patient with MEN IIb, after bilateral adrenalectomies, on maintenance steroid replacement, who underwent a neuroma resection and developed severe hypotension. There is persistent controversy regarding the general administration of perioperative “stress dose” steroids for patients with adrenal insufficiency. While the most recent literature suggests that stress dose steroids are unnecessary for secondary adrenal insufficiency, the rarer form of primary adrenal insufficiency always requires supplemental steroids, specifically hydrocortisone, when undergoing surgical procedures.

## 1. Introduction

Multiple endocrine neoplasia (MEN) type IIb is an autosomal dominant disorder causing tumors of the endocrine system, which may lead to medullary thyroid cancer and pheochromocytomas [1]. Patients can also present with neuromas of the mucosa including the lips, tongue, and intestines, often requiring multiple surgical procedures with an increased risk of anesthetic complications [2, 3]. We report a patient with MEN type IIb, after bilateral adrenalectomies, on steroid replacement, who underwent a vocal cordectomy of a neuroma and subsequently developed severe hypotension.

The patient has provided written consent to report this case.

## 2. Case Report

A 27-year-old, 68-kilogram, male patient, with a history of MEN type IIb resulting in metastatic medullary thyroid cancer and pheochromocytomas, presented to surgery for excision of a vocal cord neuroma. He denied any cardiac history and reported adequate exercise tolerance. His surgical history was significant for bilateral adrenalectomies (5 years before) and a total thyroidectomy. His medications included hydrocortisone (15 mg in the morning and 10 mg in the evening), fludrocortisone 0.05 mg daily, and levothyroxine 137 mcg daily. The patient stated that he took his usual medications for thyroid and steroid replacement the morning of surgery. Preoperatively, his heart rate was 106 bpm and blood pressure was 115/68. The rest of his vitals and lab work were within normal limits. After placement of standard monitors, the patient was induced with propofol 120 mg, rocuronium 50 mg, and fentanyl 75 mcg. A 5.5 laser endotracheal tube was placed with a C-MAC for superior visualization in order to avoid traumatizing the neuromas during intubation. An arterial line was not placed. Dexamethasone 10 mg was administered prophylactically for both postsurgical airway edema and post-op nausea and vomiting. Ten minutes into the case, the patient developed persistent hypotension: approximately 15 minutes of systolic blood pressure < 70 mmHg and mean arterial pressure < 50 mmHg despite fluid boluses (500 mL of 5% albumin) and multiple doses of ephedrine (50 mg in divided doses), phenylephrine (300 mcg in divided doses), calcium (1 gram), norepinephrine (24 mcg in divided doses), and vasopressin (6 units in divided doses). The end tidal concentration of desflurane never exceeded 4.4% and examination of the patient did not reveal any rashes or urticaria, nor was there an increase in airway pressures. Hydrocortisone 100 mg IV was administered, and an arterial line was placed. Within 10 minutes, blood pressure values returned to normal, and the procedure was completed without further complications. The patient was extubated and taken to the recovery room in stable condition.

## 3. Discussion

While the etiology and resolution of the intraoperative hypotension in this case report cannot conclusively be attributed to our decisions regarding administration of stress dose steroids, we felt that the clinical scenario provided an opportunity to review and call attention to the current evidence regarding steroid replacement practices for surgical procedures.

Glucocorticoids are produced in the zona fasciculate of the adrenal cortex. Its secretion is regulated by a negative feedback loop to the hypothalamus and pituitary gland, collectively known as the hypothalamic-pituitary-adrenal (HPA) axis. Cortisol, the principle glucocorticoid, has important functions including metabolism, immune activity, wound healing, catecholamine production, and maintenance of cardiovascular stability [4]. It is also known as the “stress” hormone because it is secreted in higher amounts when the body is faced with physiological and even psychological challenges.

Decreased cortisol secretion can be caused by adrenal gland dysfunction, known as adrenal insufficiency. While the most common form of adrenal insufficiency is from exogenous administration of corticosteroids (e.g., treatment of rheumatoid arthritis), direct damage to the adrenal glands themselves is important to consider (primary adrenal insufficiency) which includes adrenalectomies for pheochromocytomas.

Cortisone replacement with the synthetic drug hydrocortisone is the standard therapy for chronic adrenal insufficiency [5]. However, maintenance therapy to treat the physiological requirements of daily life differs from the need to predict the dose necessary for the body to respond to acute stressors such as surgery. While the literature appears to consistently support the practice of continuing a patient's maintenance doses of steroids throughout the surgical period, the administration of supplemental “stress dose” steroids has elicited much controversy and conflicting evidence.

In a 2001 review, Jabbour recommended that patients who had received more than 20 mg of prednisone daily (or equivalent) for more than 3 weeks within the previous year should be considered incapable of mounting the usual cortisol stress response and as such should be given 100 mg of hydrocortisone at induction of anesthesia [6]. Another review by Salem et al. provided more guidance by suggesting greater doses of hydrocortisone depending on the nature of surgery [7] while Coursin and Wood took the further step of recommending specific doses of hydrocortisone, taking into account not only the severity of surgery but also the illness of the patient [4].

On the contrary, in their review of the literature, Brown and Buie concluded that patients provided with just their usual maintenance steroid doses tolerated major surgery with no evidence of complications related to adrenocortical insufficiency [8]. Tasch agreed with this conclusion but opined that lower stress doses may overall be harmless and are justified by the mitigation of the rare but potentially catastrophic incidences of intraoperative hypotension [9].

More current reviews also support the claim that patients on chronic steroid therapy do not require stress dose steroids for surgery [10–12]. Kelly and Domajnko suggest that the only time additional steroids should be considered is when all other causes of perioperative hypotension have been ruled out [12].

Therefore, our initial decision not to administer hydrocortisone to our patient appeared to reflect current evidence. We did administer dexamethasone, but this was primarily to minimize postoperative airway edema at the request of the surgeon and for the well-established purpose of prevention of postoperative nausea and vomiting [13]. As described above, however, our patient became severely hypotensive despite the administration of multiple and potent vasopressors. Given the nature of the procedure, the lack of blood loss and fluid shifts, the general health of the patient (not septic), normal anesthetic doses, and lack of evidence for anaphylaxis, we considered adrenal insufficiency as a possible etiology for the refractory hypotension. Therefore, despite our administration of dexamethasone at a glucocorticoid equivalency of greater than 200 mg hydrocortisone [14], we administered an additional dose of hydrocortisone which appeared to stabilize the patient's blood pressure.

Continuing with the hypothesis of hypotension due to adrenal suppression, it is important to note that this patient suffered from* primary* adrenal insufficiency, which is the most infrequent cause of adrenal insufficiency (prevalence of 40–110 cases/million) [4]. In other words, our patient's adrenal function was not simply suppressed, but it was in essence* absent*. The literature surrounding the controversy of perioperative stress dose steroids typically focuses on secondary (ACTH dependent, e.g., pituitary depression) or the most common tertiary (hypothalamic/pituitary depression, e.g., chronic corticosteroid therapy) adrenal insufficiency [4]. In fact, of the reviews cited above, only Marik and Varon [10] and de Lange and Kars [11] make a brief statement concerning corticosteroid supplementation for patients suffering from primary adrenal insufficiency, both concluding that it is indicated in the perioperative period. Other reports of stress dose steroid management for patients with primary adrenal insufficiency are scarce and restricted to specific populations [15] and case reports [16], although steroid supplementation has been advised in these instances. Furthermore, in our case report, the apparent superior effect of hydrocortisone over dexamethasone is supported by a recent clinical practice guideline concerning the management of primary adrenal insufficiency, which states that hydrocortisone is the preferred treatment for an adrenal crisis while dexamethasone should only be considered as a last resort [17]. Intravenously injected hydrocortisone has an effect within one hour of administration [18] and is the stress steroid of choice for primary adrenal insufficiency according to multiple reviews in the literature [10, 19].

In conclusion, patients with adrenal insufficiency can pose a great anesthetic challenge. The administration of perioperative stress dose steroids to patients on maintenance doses of steroids has been a subject of much debate, but the most current literature suggests that this practice is unnecessary in most circumstances. Our case, however, should remind clinicians that adrenal insufficiency should remain on the differential for hypotension when all other causes have been ruled out and that in such situations the administration of supplemental steroids may be the treatment of choice. Primary adrenal insufficiency is rare, but in such cases stress dose steroids are always indicated, specifically hydrocortisone. Further studies should focus on perioperative implications and management of adrenal insufficiency in this specific population.
